# Few *Plasmodium falciparum* merozoite ligand and erythrocyte receptor pairs show evidence of balancing selection

**DOI:** 10.1016/j.meegid.2019.02.004

**Published:** 2019-04

**Authors:** Lynette Isabella Ochola-Oyier, Kevin Wamae, Irene Omedo, Christabel Ogola, Abneel Matharu, Jean Pierre Musabyimana, Francis K. Njogu, Kevin Marsh

**Affiliations:** a KEMRI-Wellcome Trust Collaborative Programme, P.O. Box 230, 80108 Kilifi, Kenya; b Centre for Biotechnology and Bioinformatics, College of Biological and Physical Sciences, Chiromo Campus, University of Nairobi, P. O. Box 30197, Nairobi, Kenya

**Keywords:** Malaria, Erythrocyte, Receptor, Ligand, Selection

## Abstract

Erythrocyte surface proteins have been identified as receptors of *Plasmodium falciparum* merozoite proteins. The ligand-receptor interactions enable the parasite to invade human erythrocytes, initiating the clinical symptoms of malaria. These interactions are likely to have had an evolutionary impact on the genes that encode the ligand and receptor proteins. We used sequence data from Kilifi, Kenya to detect departures from neutrality in a paired analysis of *P. falciparum* merozoite ligands and their erythrocyte receptor genes from the same population.

We genotyped parasite and human DNA obtained from 93 individuals with severe malaria. We examined six merozoite ligands EBA175, EBL1, EBA140, MSP1, Rh4 and Rh5, and their corresponding erythrocyte receptors, glycophorin (Gyp) A, GypB, GypC, band 3, complement receptor (CR) 1 and basigin, focusing on the regions involved in the ligand-receptor interactions.

Positive Tajima's D values (>1) were observed only in the MSP1 C-terminal region and EBA175 region II, while negative values (<−1) were observed in EBL-1 region II, Rh4, basigin exons 3 and 5, CR1 exon 5, Gyp B exons 2, 3 and 4 and Gyp C exon 2. Additionally, ebl-1 region II and basigin exon 3 showed extreme negative values in all three tests, Tajima's D, Fu & Li D* and F*, ≤ − 2.

A large majority of the erythrocyte receptor and merozoite genes have a negative Tajima's D even when compared with previously published whole genome data. Thus, highlighting EBA175 region II and MSP1–33, as outlier genes with a positive Tajima's D (>1). Both these genes contain multiple polymorphisms, which in the case of EBA175 may counteract receptor polymorphisms and/or evade host immune responses and in MSP1 the polymorphisms may primarily evade host immune responses.

## Highlights

• Merozoite ligands and erythrocyte receptor pairs show largely negative summary statistics.
• Basigin exon 3 and EBL-1 region II showed extreme negative Tajima's D values.
• EBA175-Glycophorin A, EBL1-Glycophorin B, MSP1-band 3 and Rh5-Basigin are highlighted as gene pairs of interest.

## 1 Introduction

The impact of malaria on human evolution is evident from the signatures of selection in genes encoding erythrocyte proteins in human populations from malaria endemic regions. Examples of host erythrocyte polymorphisms that are at a high frequency in malaria endemic regions include the classic case of the Duffy negative blood group which protects against *Plasmodium vivax* infection (Miller et al., 1976), sickle cell trait that protects against severe malaria (Kwiatkowski, 2005; Williams et al., 2005) and more recently a Dantu variant that also showed protection against severe malaria (Leffler et al., 2017). These polymorphisms have most likely been selected to high frequency because of the strong selective pressure provided by the pathological complications of malaria.

Evolutionary selection pressures are also evident in *Plasmodium falciparum* genes, particularly those involved in the erythrocytic stages of the parasite's life cycle that cause disease pathogenesis. Erythrocyte invasion is a complex process and in the case of *P. falciparum*, the dominant malaria parasite in Africa, involves multiple merozoite ligand - erythrocyte receptor interactions. Previous studies have shown evidence of selection in several merozoite antigens known to be involved in the invasion of erythrocytes (Hughes, 1992; Polley and Conway, 2001; Baum et al., 2003; Rayner et al., 2005; Polley et al., 2007; Tetteh et al., 2009; Ochola et al., 2010), presumably to allow the parasite to escape host immunity by allele-specific mechanisms, such that an immune response to one allele does not protect against a different allele (Crewther et al., 1996; Polley et al., 2007). However, parasite ligands may also need to adapt to tolerate erythrocyte receptor diversity to maintain interactions and facilitate erythrocyte invasion.

Despite decades of work, relatively few *P. falciparum* ligand-receptor interactions have been defined*;* erythrocyte binding antigen (EBA) 175 and Glycophorin (Gyp) A (Sim et al., 1994) was the first interaction defined and the crystal structure of EBA175 region II only recently described (Tolia et al., 2005). In the subsequent two decades, another five interactions were identified, EBA140 and Gyp C (Lobo et al., 2003), merozoite surface protein (MSP) 1 and band 3 (Goel et al., 2003), erythrocyte binding ligand (EBL)- 1 and Gyp B (Mayer et al., 2009), reticulocyte binding homologue (Rh) 4 and Complement Receptor 1 (CR1) (Tham et al., 2010), and most recently Rh5 with Basigin (BSG) (Crosnier et al., 2011).

Genetic variation in the glycophorins, in particular Gyp A, has been studied in the most detail. Multiple mutations in Gyp A exon 2 have shown evidence of being under selection (Baum et al., 2002; Wang et al., 2003; Ko et al., 2011), potentially due to its interaction with EBA175. A recent study identified Gyp A exon 3 as the critical glycosylated region that interacts with EBA175 region II (Salinas et al., 2014). The S-s-U- Gyp B phenotype is characterized by a lack of Gyp B protein expression on the erythrocyte surface, due to a deletion in exons 2 to 5, and these erythrocytes were relatively resistant to invasion by some strains of *P. falciparum* (Tarazona-Santos et al., 2011). A well-known but rare Gyp C polymorphism, the Gerbich negative phenotype (a deletion in exon 3) (Colin et al., 1989; Wilder et al., 2009), renders erythrocytes partially resistant to invasion, since the parasite is unable to utilise the merozite ligand EBA140 (Maier et al., 2003).

Binding regions have also been identified in the more recently defined erythrocyte receptors, Band 3, CR1 and BSG. The combined 5ABC and 6A ectodomain regions of band 3, encoded by exons 17 and 18 respectively, interact with the MSP1-42kDa processed product through the 19 kDa C-terminal domain (Li et al., 2004). PfRh4 binds to complement-control-protein (CCP) repeats 1–3 in the ectodomain portion of CR1 (Tham et al., 2011) encoded by exons 2, 3, 4 and 5 (Vik and Wong, 1993). BSG also termed the Ok blood group antigen, has two splice isoforms, BSG-L (long) and BSG-S (short). BSG-S has two immunoglobulin superfamily (IgSF) domains, which are both required for binding to Rh5 (Crosnier et al., 2011). Wright et al. (2014) described the crystal structure of Rh5 in a complex with BSG and the BSG protein structure was encoded by exons 4, 5, 6 and part of exon 7.

Erythrocytes are the most abundant cells in human blood and potentially act as decoys for viruses and bacteria, to bind and clear them, while they are a host for the malaria parasite, these pathogens present opposing selection pressures (Gagneux and Varki, 1999). While variation in the parasite invasion ligands and their erythrocyte receptors have been the subject of frequent study, there have been very few studies of variation in parasite ligands and their cognate receptors in the same individuals, yet it is exactly these kinds of studies that may reveal genes of interest. Furthermore, most of the previous merozoite ligand and erythrocyte receptor diversity studies were conducted in individuals who either had uncomplicated malaria or no malaria at all at the time of sampling. In this paper, we examine individuals with severe malaria due to its various syndromes including impaired consciousness (also referred to as cerebral malaria), severe malarial anemia and respiratory distress (Marsh et al., 1995) and under the hypothesis that this distinct disease phenotype could skew the population towards a particular ligand-receptor genotype. We examined this using the merozoite ligand - erythrocyte receptor interactions. Individuals and their infecting *P. falciparum* parasite population were genotyped to determine whether the polymorphisms in erythrocyte receptors (Gyp A, Gyp B, Gyp C, band 3, CR1 and BSG) and their merozoite ligands (EBA175, EBL1, EBA140, MSP1, Rh4 and Rh5, respectively) show a skewed population genetic summary statistic when analysed at the same time point. The study focused on the polymorphisms in regions involved in the ligand-receptor interaction, an approach that has not been taken before for Gyp C, CR1, band 3 and BSG and for all the above genes in a paired analysis.

## 2 Materials and methods

### 2.1 Population sampled

Parasite and human DNA were obtained from 93 unrelated children aged 0 to 12.3 years (mean age, 2.98) who were admitted at Kilifi County Hospital's High Dependency Unit (HDU) with severe malaria (mean parasitemia −34,830 parasites/μl, range 102–1,171,800 parasites/μl) in 2001. Severe malaria was defined as individuals who were parasite positive with any one of the following: a Blantyre coma score of <3, a haemoglobin level of <5 g/dl or respiratory distress or deep breathing (Marsh et al., 1995). This study was reviewed and approved by the Scientific Steering Committee and the Ethics committee of the Kenya Medical Research Institute.

### 2.2 DNA extraction, amplification and sequencing

**Fig. 1:**
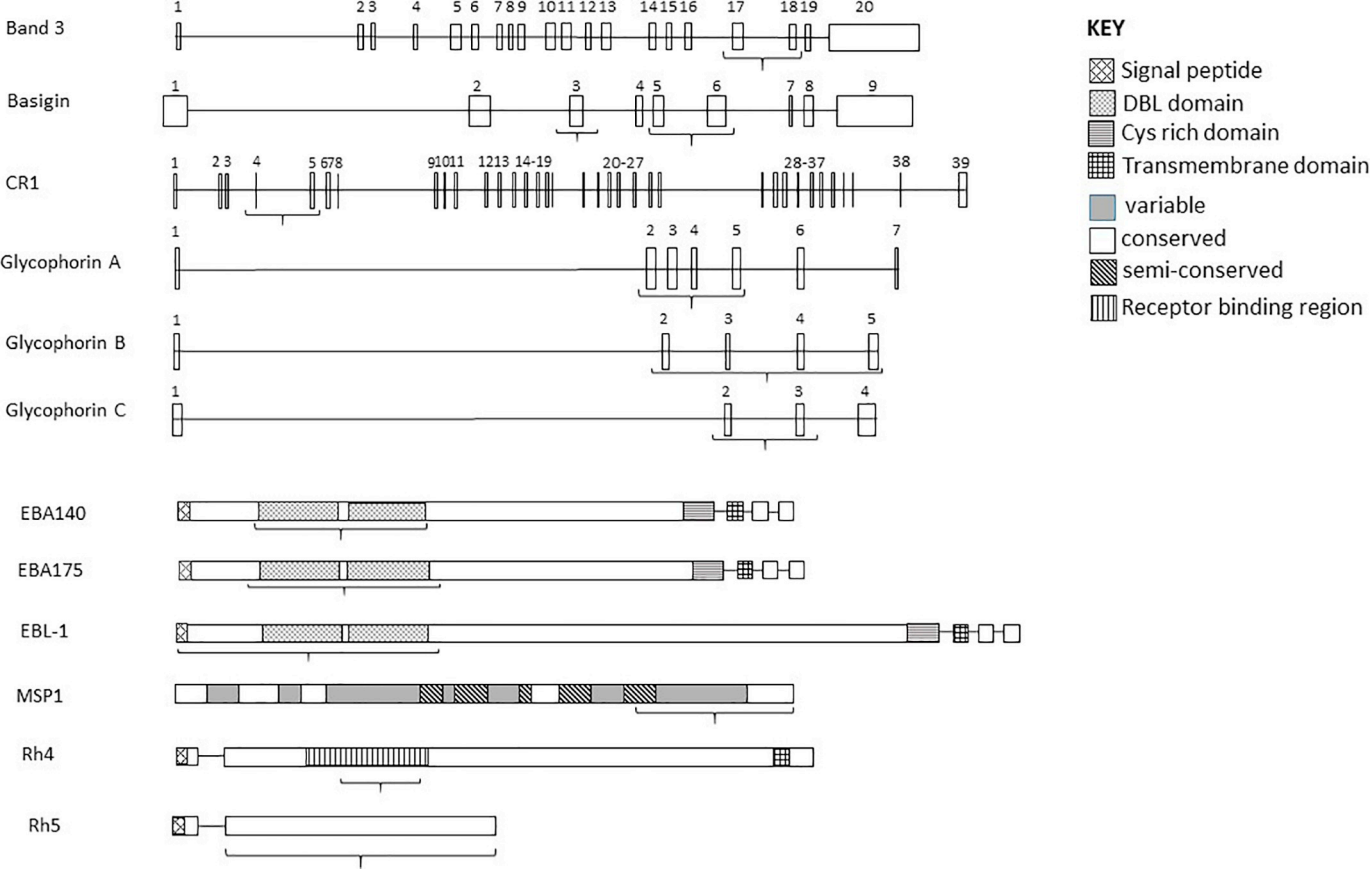
Scheme of the six erythrocyte receptor and merozoite ligand genes. The curly brackets indicate the regions of the genes identified as being involved in the receptor-ligand interaction that were sequenced. The scheme indicates the positions of the exons and the lengths are relative to differentiate the bigger from the smaller genes. These regions were amplified and sequenced to determine whether they were under selection.

We examined the genes of the six merozoite ligands, EBA175, EBL1, EBA140, MSP1, Rh4 and Rh5, and their respective erythrocyte receptors, GypA (NG_007470), GypB (NG_007483), GypC (NG_007479), band 3 (NG_007498), CR1 (NG_007481) and BSG (NG_007468) (Fig. 1). Genomic DNA was previously extracted from packed frozen erythrocytes using the QIAamp DNA Blood Mini Kit, according to the manufacturer's instructions (QIAGEN, UK). All 12 genes were amplified using High Fidelity Taq polymerase (Roche) (details of regions amplified (Fig. 1) and primers used are shown in Supplementary Table 1). PCR products were visualised on 1% agarose gels, purified using ethanol precipitation or EXOSAP-IT (Affymetrix) and directly sequenced using the PCR primers and additional sequencing primers (Supplementary Table 1), BigDye terminator chemistry v3.1 (Applied Biosystems, UK) and an ABI 3130xl capillary sequencer (Applied Biosystems, UK). PCR products with poor quality sequences were reamplified and resequenced. Sequences were assembled, edited, and aligned using SeqMan and MegAlign software (Lasergene 12; DNASTAR) along with Bioedit (Hall, 1999) and MEGA (Tamura et al., 2007). All singleton SNP loci were confirmed by independent reamplification and resequencing of the relevant samples. Positions of the sequences that showed mixed or superimposed nucleotides (peak within a peak) were marked with IUPAC ambiguity codes. For the erythrocyte receptors they were marked as heterozygous and for the merozoite ligands as a mixed infection, in the latter the mixed infections were excluded from the SNP and haplotype frequencies. The Gyp A and Gyp B sequences were aligned to previously published sequences in NCBI to verify the sequences we obtained, since the two genes share high sequence homology.

### 2.3 DARC and *Plasmodium vivax* genotyping

Initially, the samples were genotyped at the −33 T/C GATA-1 promoter mutation of the Duffy Antigen Receptor for Chemokines (DARC) locus using a Restriction Fragment Length Polymorphism (RFLP) analysis including the primers and PCR conditions adopted from Weppelmann et al. (2013), and the *Sty*I restriction endonuclease. The samples were later capillary sequenced at both the GATA-1 promoter region and the aspartate to glycine amino acid substitution at codon 42 which determines the Fy^b^ and Fy^a^ blood-group antigens, respectively. A *P. vivax* PCR targeting the 18S ribosomal RNA was also conducted to determine the prevalence of *vivax* infections in the Kilifi population. The *P. vivax* control DNA was a kind donation from Dr. Julian Rayner's laboratory.

### 2.4 Sequence cluster analysis

All the sequences were aligned in Clustal Omega v1.2.0 (Sievers et al., 2011). Following alignment, the sequences were trimmed to the same length and any short sequences excluded to obtain the largest number of SNPs for the generation of haplotypes. The positions of the SNPs were noted and used to determine the haplotypes per sample. The trimmed multiple alignment files were translated into amino acid sequences, exported as FASTA files and clustered at 100% identity in usearch v. 7.0.1001 (Edgar, 2010). The number of clusters generated were then taken as the number of haplotypes for each of the genes.

### 2.5 Population genetics statistical tests

The allele frequency distribution indices, Tajima's D and Fu and Li’s D* and F*, were computed using DnaSP v5.10 software (Rozas et al., 2003). Tajima's D computes the differences between two estimators of theta, based on the number of segregating sites and the average number of nucleotide differences (Tajima, 1989). Fu and Li’s D test statistic calculates the differences between the observed number of singletons (mutations appearing only once among the sequences), and the total number of mutations (Fu and Li, 1993). Fu and Li’s F test statistic considers the differences between the number of singletons and the average number of nucleotide differences between pairs of sequences (Fu and Li, 1993). For the *p* values DnaSP calculates the confidence limits of D (two-tailed test) and assumes that the statistic follows a beta distribution. The erythrocyte receptor data were read as unphase (or genotype) data files to factor in diploid individuals and the files included the IUPAC nucleotide ambiguity codes to represent heterozygous sites.

### 2.6 Ligand-receptor protein structures

Only the receptor-ligand interacting complex between Rh5 and BSG has a published three-dimensional structure (Chen et al., 2014; Wright et al., 2014). Both protein crystal structures (4UOQ) were downloaded in the Protein Data Bank (PDB) (http://www.rcsb.org/) format and were viewed using Pymol v. 1.7.2.1 (www.pymol.org). The protein structure for Rh5 encompass amino acid residues 160–242 and 302–504 and for BSG residues 23–203. Using our dataset, the Rh5 and BSG polymorphic sites were mapped onto their protein structures in Pymol, to determine the location of the identified polymorphisms in three-dimension and whether these polymorphic sites were found in the binding regions of either protein.

## 3 Results

### 3.1 Merozoite ligand and erythrocyte receptor diversity

We obtained between 26 and 49 good quality sequences for eba140, eba175, ebl1 region I, ebl1 region II, msp1–19, the 5′ end of msp1–42, rh4 and rh5 (Table 1). Only the regions sequenced for EBA175 and MSP1 did not contain any singleton loci, 3 genes had indels and ebl-1 regions I and II both contained stop codons (Table 1). The ebl-1 region I stop codons were at positions 39 and 185 (due to a 5 Thymidine insertion) and in region II codon 664, with a frequency of 28% (total = 25), 72% (total = 25) and 15% (total = 38), respectively. Only 12% total = 25) of the samples did not have a stop codon at either positions 39 or 185. It is worth noting that both mutations are not mutually exclusive. Nucleotide diversity (π) in these genes was higher than the erythrocyte receptor genes, with only 2 gene regions being <0.001. MSP1–19 kDa had the highest π value of 0.006, while EBL1 region II had the lowest value, 0.00052 (Table 1). Additionally, there were several haplotypes identified for each gene using cluster analysis and the dominant haplotypes were INGKK, KEEKKSISENK/KEKEKPISENK, QFF*, IYGKHN, EKSNGL, KDD, NKK and DYHYIK for eba140, eba175, ebl1 region I, ebl1 region II, msp1–19, the 5′ end of msp1–42, rh4 and rh5, respectively (Table 2). Moreover, each merozoite gene had at most four dominant haplotypes (present at a frequency of >10%) except for ebl-1 region II, which only had 1 dominant haplotype. >40% of the infections were from the dominating haplotype for each ligand, except for EBA175 region II in which diversity was more extensive (only 13% of the infections were from the two dominating haplotypes) (Table 2). In contrast, we obtained between 22 and 84 good quality sequences for the different exons for band 3, BSG, CR1, Gyp A, Gyp B and Gyp C (Table 1). These genes were less polymorphic than the merozoite ligands, contained more singletons and all the exons genotyped revealed one dominant haplotype (>90%) (Table 2). This reduced diversity was reflected in the π values, which were overall much lower than those for the merozoite ligands (six of the eleven regions sequenced were < 0.001) and ranged from 0.00007 for band 3 exon 18 to 0.02 for Gyp A exon 2 (Table 1). The greatest diversity was observed in Gyp A exon 2, while Gyp A exons 4 and 5 did not contain any polymorphic loci. Gyp A exon 3 was difficult to resolve as there was a high mix of Gyp A and Gyp B sequences and thus the sequences were not assembled for analysis. Of a total of eight Gyp A exon 2 haplotypes, three were present at a frequency of >10%. Gyp B exons 3 and 4 presented two haplotypes at >10% frequency, from a total of four and five haplotypes, respectively.

**Table 1:**
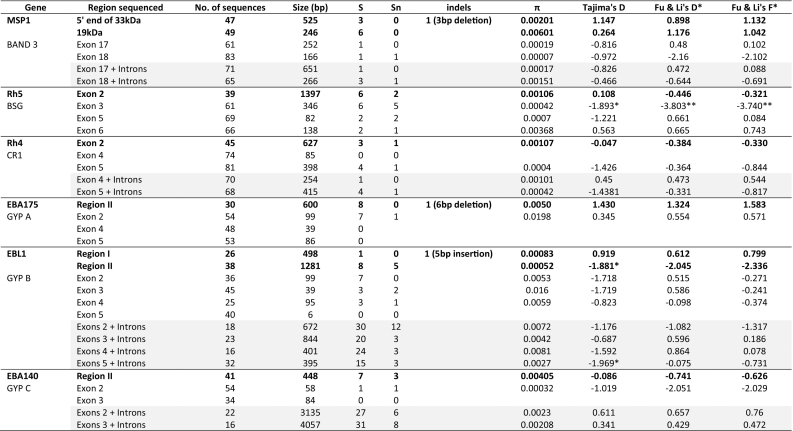
Summary of DNA polymorphisms and population genetic statistics of the merozoite ligands and erythrocyte receptor genes.

^*⁎*^*p* < .05; ^*⁎⁎*^*p* < .02; S- segregating sites; Sn- singletons; π - nucleotide diversity; Gyp A exon 3 was sequenced but the data was a mix of Gyp A and Gyp B sequences and was unresolvable, ebl-1 region I contained 2 stop codons and ebl—1 region II contained 1 stop codon, Bold text represent the merozoite ligands, grey shade highlights the exon+ intron analyses.

**Table 2:** The frequency of the merozoite ligand and erythrocyte receptor haplotypes.

Gene	Region sequenced	Haplotypes	Frequency, n (%)	Gene	Exons sequenced	Haplotypes	Frequency, n (%)
EBA140	REGION II (*n* = 42)	INGKK	20 (47.6)	Gyp C	exon 2	PP	53 (98.2)
	codons 185,239,257,	VSGKK	8 (19.1)		codon 20	PR	1 (1.9)
	261,285	VSGTK	6 (14.3)				
		VNGRE	3 (7.1)				
		INGRE	2 (4.8)				
		ISGKK	1 (2.4)				
		VNGKK	1 (2.4)				
		VSVTK	1 (2.4)				
EBA175	REGION II (*n* = 30)	KEEKKSISENK	4 (13.3)	Gyp A	exon 2 (*n* = 54)	TT,DG,SS	30 (56.6)
	codons 226, 274, 279, 286,	KEKEKPISENK	4 (13.3)		codon 23	SS,GG,SS	7 (13.2)
	388, 390, 401,402,403,	KEEKNSISKNK	3 (10)			TT,GG,SS	7 (13.2)
	404,405	EKEKKPISENK	2 (6.7)			TT,EE,SS	3 (5.7)
		KEEEKPISENK	2 (6.7)			TT,DD,SS	2 (3.8)
		KEEKKPISENK	2 (6.7)			TT,DG,SF	2 (3.8)
		KEEKNSKM	2 (6.7)			TT,DD,SF	1 (1.9)
		KKEENSKM	2 (6.7)			SL,GG,SS	1 (1.9)
		KKEKKPISENK	2 (6.7)				
		KKEKNSISKNK	2 (6.7)				
		EEKEKSISENK	1 (3.3)				
		KEEEKPISKNK	1 (3.3)				
		KEEKNSISENK	1 (3.3)				
		KEKENPISENK	1 (3.3)				
		KKEKNSNM	1 (3.3)				
EBL1	Region I (*n* = 26)	QFF*	15 (57.7)	Gyp B	exon 2 (*n* = 36)	EE,LL,TT,EE	33 (91.7)
	codons 39,187,188,189,	QLSK	4 (15.4)		codons 13, 20, 23,	EE,WW,SS,GG	1 (2.8)
	191,192,193	*LSK	4 (15.4)		24	EE,LW,SS,EG	1 (2.8)
		*FF*	3 (11.5)			EK,LL,TT,EE	1 (2.8)
	Region II (*n* = 38)	IYGKHN	32 (84.2)		exon 3 (*n* = 45)	EE,TT,LL,RR,FF,TT	36 (80)
	codons 342,347,658,	ICGKHN	2 (5.3)		codons 47, 48, 51,	EE,MM,LL,RR,FF,TT	5 (11.1)
	664,690	IYG*H*	2 (5.3)		54,55, 56	EE,TM,LL,RR,FF,TT	3 (6.7)
		IYCKYN	1 (2.6)			KK,GG,FF,LL,LL,SS	1 (2.2)
		MYGKHN	1 (2.6)		exon 4 (*n* = 25)	AA,II,SS	16 (64)
					codon 59, 66, 84	AA,II,TT	5 (20)
						AA,II,ST	2 (8)
						AA,MM,SS	1 (4)
						TT,II,SS	1 (4)
MSP1	42 kDa fragment (*n* = 47)	KDD	19 (40.4)	Band 3	exons 17 & 18 contained 1 synonymous SNP		
	codons 1472,1516,1533	KDN	15 (31.9)				
		DD	6 (12.8)				
		HD	4 (8.5)				
		DN	3 (6.4)				
	19 kDa fragment (*n* = 49)	EKSNGL	22 (44.9)				
	codons 1620,1667,1675,	QKSNGL	10 (20.4)				
	1676,1677,1692	QKSNGF	8 (16.3)				
		ETSSRL	3 (6.1)				
		EKSSRL	2 (4.1)				
		QKNNGL	2 (4.1)				
		QTSNGF	1 (2)				
		QTSNRL	1 (2)				
Rh4	exon 2 (n = 45)	NKK	25 (55.6)	CR1	exon 5 (*n* = 81)	TT	77 (96.3)
	codons 435,438,500	NQK	16 (35.6)		codon 173	TA	3 (3.7)
		KQK	2 (4.4)				
		KKK	1 (2.2)				
		NQI	1 (2.2)				
Rh5	exon 2 (*n* = 39)	DYHYIK	19 (48.7)	Basigin	exon 3 (*n* = 61)	AA,II,DD,AA,LL	56 (91.8)
	codons 133,147,148,203,	DYHYIN	6 (15.4)		codons 28, 93,115,	AA,II,DD,AA,LV	1 (1.6)
	410,429	DHDYIN	5 (12.8)		130,135	AA,II,DD,PP,LL	2 (1.6)
		DHDYIK	4 (10.3)			AA,II,DY,PP,LL	3 (1.6)
		DYHCIK	2 (5.1)			AA,IM,DD,AA,LL	4 (1.6)
		DYHCIN	1 (2.6)			AE,II,DD,AA,LL	5 (1.6)
		DYHYMK	1 (2.6)		exon 5 (*n* = 69)	LL	68 (98.5)
		NYHCIN	1 (2.6)		codon 90	FF	1 (1.5)
					exon 6 (*n* = 66)	EE	65 (98.5)
					codon 146	DD	1 (1.5)

CR1 exon 4 and Gyp B exon 5 did not contain any SNPs.

### 3.2 Merozoite ligand- erythrocyte receptor haplotype pairs

A paired analysis of haplotypes revealed that the merozoite ligands had a higher number of haplotypes in the population compared to those observed for the erythrocyte receptors. Region II of EBA175 and EBL-1 had lower π values than their receptors GypA and GypB, respectively (Table 1). Even though EBA175 had a lower π value (Table 1) it was even more diverse with 15 haplotypes compared to Gyp A exon 2 that had about half the number of haplotypes (Table 2). On the other hand, EBL-1 region II presented 5 haplotypes and a corresponding number of haplotypes was observed in Gyp B exons 2, 3 and 4. Large differences in the number of haplotypes were also observed, of note, there were a total of eight EBA140 haplotypes and only two haplotypes for Gyp C exon 2 and the same number between Rh5 and BSG exons 5 and 6 (two haplotypes each). We could not assess whether some merozoite ligand - erythrocyte receptor haplotype pairs were preferred over others, since most of the erythrocyte receptor genes in this population either had very few polymorphic sites or singletons and therefore the population was dominated, >90%, by one haplotype (Table 2). We examined band 3, BSG, CR1 and Gyp C in Ensemble genome browser (https://www.ensembl.org/index.html) to further explore genetic variation in these genes from the human 1000 genome project from 9 populations, Africa, Americas, Ashkenazi Jewish, East Asian, South Asian, Finnish, Non-Finnish European and Other. Notably, we did not come across the SNPs identified in this study in the whole genome data, but identified other rare variant loci that were primarily 100% the ancestral allele. For instance, in BSG, rs145916692 in codon 95, exon 5, in CR1, rs371731619 in codon 159, exon 4 and in Gyp C rs143216051 in codon 42, exon 3 was 98% the ancestral allele in the African population and 100% the ancestral allele in the other populations. We identified different loci in our study such as codon 90 in BSG, a singleton in Gyp C in exon 2 and no SNPs in exon 3 and in band 3 most of the 1000 genome variants were not in the region studied and were upstream of exons 17 and 18. Thus, it appears that most loci in the erythrocyte receptor regions are potentially rare variant sites.

Most of the variation observed was in the merozoite antigens as they contained multiple polymorphic loci and a larger number of haplotypes circulating in the population (Table 2). The prevalent haplotypes in the paired analysis represented the dominant haplotype in the population. The merozoite antigen haplotypes were compared with previous published data from a more recent time point (2007/2008) of children with uncomplicated malaria (Ochola-Oyier et al., 2016) and for all 6 merozoite antigens similar haplotypes were still circulating in the Kilifi population as the dominant haplotypes. However, in EBA140 we observed two different high frequency haplotypes in the severe malaria population of INGKK and VSGKK, with VSGTK presenting at a lower frequency in comparison to the more recent data where it was the dominant haplotype. For Rh5, we observed an excess of the YH compared to the HD haplotype (when only considering the high frequency polymorphic loci, codons 147 and 148) in this severe malaria population which was similar to the more recent time points in the uncomplicated, asymptomatic and complicated/severe cases and the 10-year temporal data of all infections (Ochola-Oyier et al., 2016), which also had a predominance of the YH haplotype. In these individuals with severe malaria, the parasite population does not differ from the rest of the population of parasites circulating over time in Kilifi, suggesting that at these loci it is not a specific ligand or receptor population that results in severe malaria.

### 3.3 Merozoite ligand- erythrocyte receptor pair summary statistics

**Fig. 2:**
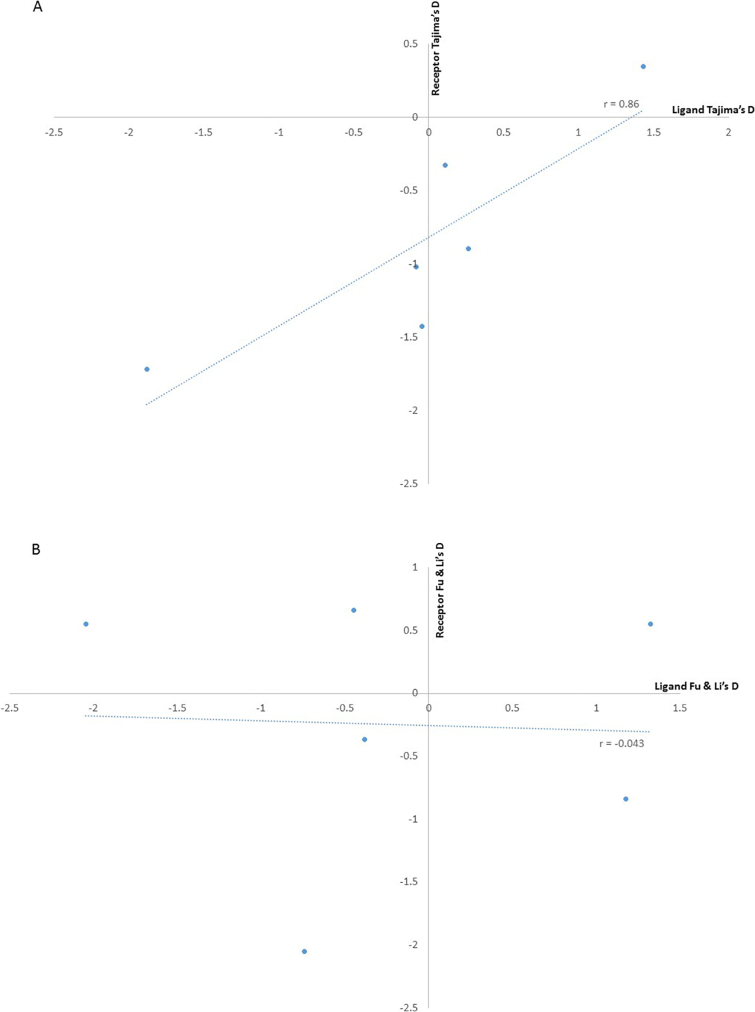

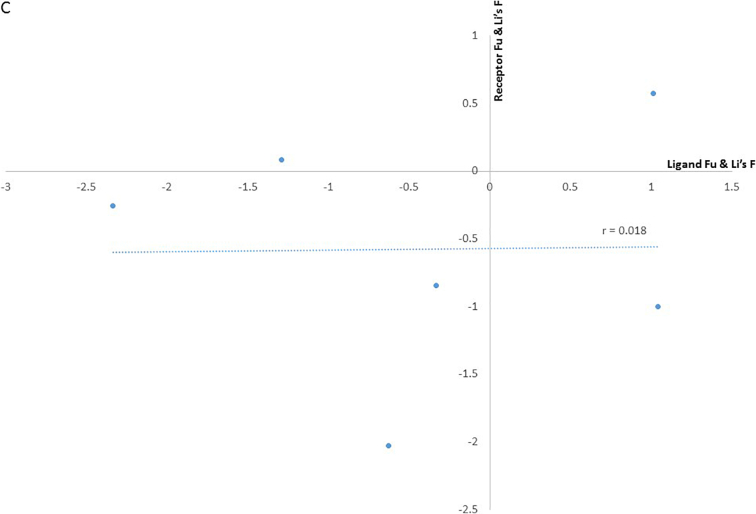
Scatter plot showing the correlations between the summary statistics indices for each erythrocyte receptor-merozoite ligand pair. The merozoite ligand Tajima's D value was plotted against its erythrocyte receptor Tajima's D value, the same was done for the Fu & Li D* and F* plots. The values used from the erythrocyte receptors with data from more than one exon were as follows: for band 3 the average of both exons 17 and 18 plotted against MSP1–19 since it is the region that primarily interacts with the receptor, BSG only exon 5 was considered since previous literature has shown altering codon 90 lowers binding (Crosnier et al., 2011) and it was plotted against Rh5, Gyp B- was an average of exons 2 and 3, similar to Gyp A for which exons 2 and 3 are considered to be involved in binding EBA175, plotted against EBL-1 regions and Gyp A exon 2 was plotted against EBA175. (A) Shows the correlation of the Tajima's D statistic for each receptor-ligand pair, (B) the correlation of the Fu & Li’s D* statistic for each receptor-ligand pair and (C) is the correlation of the Fu & Li’s F* statistic for each receptor-ligand pair. The Pearson product-moment correlation coefficient, r, is shown on the graphs.

The majority of the ligand-receptor pairs exhibited negative summary statistics in particular, EBL-1 region II and BSG exon 3 both had extreme negative Tajima's D values of −1.881 (*p* < .05) and − 1.89, (p < .05), respectively. Similar results were obtained for Gyp B exons 2 and 3 (−1.7) and BSG exon 5 (−1.2) (Table 1). Notably, EBL-1 region II and BSG exon 3 also had negative summary statistics for Fu & Li D* and F* tests, <−2 for both tests. There is therefore an excess of low frequency mutations or singletons in these regions. In contrast, EBA175 and MSP1 were positive for all 3 test statistics with a value of ≥1 (Table 1). Thus, drawing attention to these genes, in contrast however their receptors Gyp A and band 3, respectively, fall within the neutral range. We also examined the correlation between the values of the summary statistics for the merozoite ligand and erythrocyte receptor pairs and observed no correlations using the Fu & Li D* and F* indices, (Fig. 2A and B). Though the data points are few, the Tajima's D index (Fig. 2C) showed an indication towards a strong linear relationship, using the Pearson product-moment correlation coefficient (*r* = 0.86), suggesting the index goes in the same direction for the ligand-receptor pairs. Subsequently, a positive Tajima's D value for the merozoite ligand would indicate a positive value for its receptor and vice versa.

### 3.4 Comparisons of Tajima's D with previously published data

Our study, highlights EBA175 region II (Tajima's D, 1.43) and MSP1–33 (Tajima's D, 1.15) since they had the highest positive values as genes with potential functional importance. We therefore compared the Tajima's D values from this study with those from previous studies. We first assessed the EBA175-GypA pair further since there is previously published data using the same methods of capillary sequencing. We obtained similar Tajima's D statistics (1.07) for EBA175 region II as a previous study with a similar sample size of 30, in Nigeria, from a 1800 bp region II fragment which contained 17 segregating sites and 1 singleton (Baum et al., 2003). Moreover, a study done in a similar population in Kilifi, Kenya but from a cohort of individuals in the community, also showed similar Tajima's D (1.13) results from a sample size of 39 individuals and a region II fragment size of 1730 bp, with 19 segregating sites and 2 singletons (Verra et al., 2006). Evidence of balancing selection in Gyp A exon 2 has previously been described, in Nigeria, in 33 individuals yielding a significant Tajima's D of 2.54 from 5 segregating sites in the 99 bp fragment (Baum et al., 2002). Four different populations in Kenya, two from a malaria hyperendemic area obtained significant Tajima's D values of 2.6 and 2.4 and a hyperendemic and hypoendemic region resulted in Tajima's D values of 1.9 and 1.8, respectively. The sample size for the four regions was 34, 38, 34 and 22, respectively (Ko et al., 2011). These studies did not appear to use samples from individuals infected with malaria though they were from malaria endemic regions and were more likely to represent the general population. These studies are in great contrast to the finding of our study of a Tajima's D of 0.345 from 54 samples, thus suggesting that this region of the gene is largely neutral in the severe malaria population.

An additional comparison was done using previously published whole parasite genome Tajima's D scans from 3 populations, which showed that the parasite genome is largely negative (Amambua-Ngwa et al., 2012; Mobegi et al., 2014; Ocholla et al., 2014). In a Gambian population, using the Tajima's D statistics and 2853 genes with >3 SNPs, they identified 115 genes with a Tajima's D value ≥1, 222 genes with values between 0 and 1, 788 between 0 and − 1 and 1728 genes from −1 to −2.5 (Amambua-Ngwa et al., 2012). Based on this data, ~80% of the polymorphic genes are skewed towards a negative Tajima's D, which is about 47% of the whole genome that contains ~5300 genes (Gardner et al., 2002). The top Tajima's D hits of >1 identified 18 genes in a Gambian and Guinean population (Mobegi et al., 2014), 19 genes ≥1 in a Malawian population (Ocholla et al., 2014) and 25 genes with at least 10 SNPs (Amambua-Ngwa et al., 2012) (Supplementary Table 2). We compared the Tajima's D values from this study with the previous studies and highlight EBA175 (Tajima's D = 1.43) and MSP1 (Tajima's D = 1.147) that fall within the range of the top hits from the 3 studies of whole genome scans. The study from Malawi, which is geographically closer to Kenya than the Gambia or Guinea, is the only study that identified EBA175 as a gene with a > 1 Tajima's D value. Thus, a Tajima's D value >1 is a reasonable cut off for our data.

### 3.5 Ligand and receptor protein structures

**Fig. 3:**
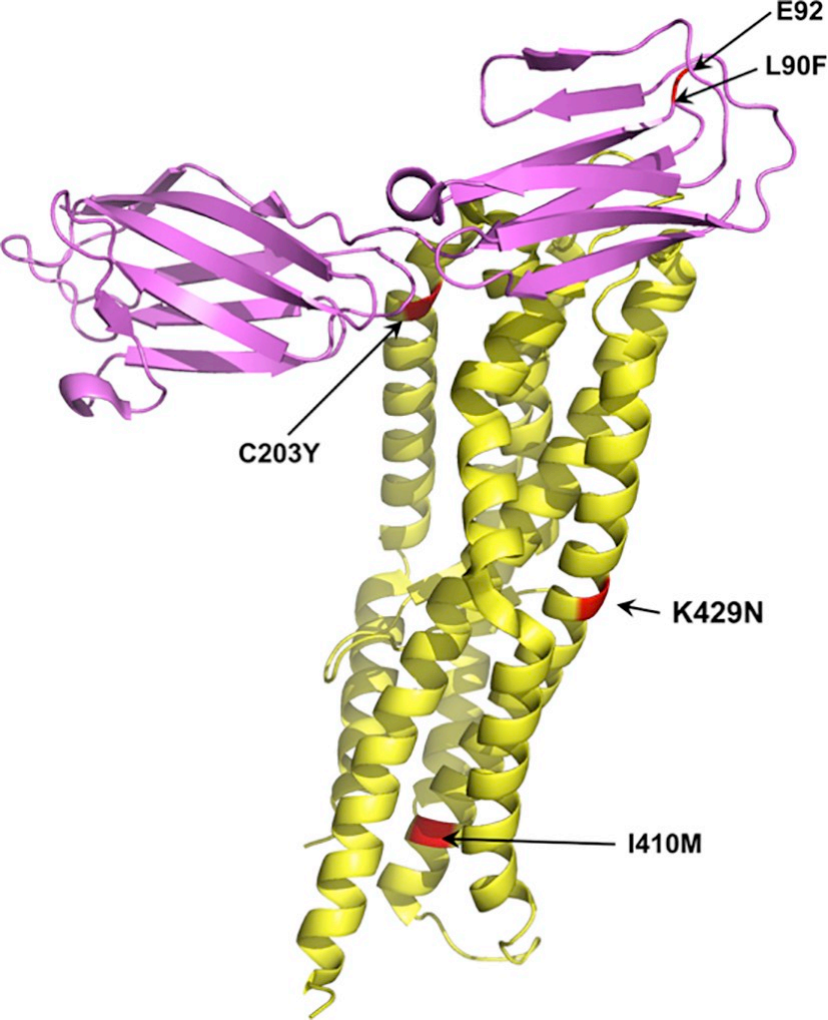
The structure of the Rh5-BSG interaction. The Rh5 structure is shown in yellow and represents amino acid residues from 160 to 242 & 302–504 and the SNPs identified in this study are highlighted in red. It is only the SNP at codon C203Y of Rh5 that is involved in the interaction with basigin, while I410M and K429N fall outside the binding region with basigin. The pink structure represents basigin from amino acid residues 23–203 with the SNP (L90F) identified in this study highlighted in black and the Ok variant locus (92) is also shown. Both codons 90 and 92 appear to be involved in the interaction with Rh5. (For interpretation of the references to colour in this figure legend, the reader is referred to the web version of this article.)

Of the seven polymorphisms identified in Rh5 only one, C203Y, fell in the region that interacts with BSG (Fig. 3). This SNP, C203Y, is next to codon 204, which has previously been shown to play a role in determining the ability *of P. falciparum* strains to invade *Aotus nancymaae* erythrocytes (Hayton et al., 2008). The rest of the Rh5 SNPs appear to fall outside the BSG binding region. One rare variant of BSG, L90F, is in the region that interacts with Rh5 and is close to the Ok variant locus (codon 92). In a previous study, OK variant negative erythrocytes were shown to be recalcitrant to invasion (Crosnier et al., 2011), but this mutation has only ever been found in Japanese individuals, and the Kilifi population was as expected, 100% wild type at codon 92.

### 3.6 DARC and *P. vivax* genotyping analysis

Though *Plasmodium vivax* is more widely distributed outside sub-Saharan Africa, we confirmed as a control that the study population was Duffy Antigen Receptor for Chemokines (DARC) negative and that *P. vivax* infections were absent. The 93 samples were all Duffy antigen negative by PCR-RFLP and this was confirmed by amplifying and sequencing 91 samples, all of which contained the −46C variant in the DARC promoter region, which silences the expression of the DARC gene. At codon 42, all the 91 samples sequenced coded for the amino acid aspartate, thus the entire population sampled expressed the FY*B^null^ allele, which confirms previous findings that most people of African descent carry the erythrocyte silent FY*B ancestral allele (Miller et al., 1976; Tournamille et al., 1995). The 18S rRNA *P. vivax* primers did not yield any PCR amplicons from all 93 samples, thus no *P. vivax* was detected in this population.

## 4 Discussion

Overall the analysis of both merozoite ligands and erythrocyte receptors showed negative summary statistics, except for MSP1-33 and EBA175 region II. Moreover, only BSG exon 3 and EBL-1 region II showed extreme negative results, ~−2 Tajima's D. These results are not surprising since, previous studies of *P. falciparum* populations using whole genome sequence data showed that the majority of genes in the parasite genome were associated with negative Tajima's D values (Amambua-Ngwa et al., 2012; Mobegi et al., 2014; Ocholla et al., 2014). These results are reflective of a historical parasite population expansion in Africa. Thus, the positive Tajima's D values (>1) observed in this study (for MSP1 and EBA175) against the backdrop of negative values from a comparison of whole genome data, are highlighted as outliers consistent with previous results that have identified these genes as being under balancing selection (Hughes, 1992; Baum et al., 2003; Verra et al., 2006).

It has also been suggested that as a much as 70–75% of amino-acid altering mutations were affected by moderate or strong negative selection in the human genome (Eyre-Walker et al., 2002; Eyre-Walker et al., 2006; Kryukov et al., 2007). Therefore, the human genome primarily exhibits negative statistics as demonstrated by the erythrocyte loci examined in this study. Since the malaria parasite has a long history of coevolution with humans observing the negative values for both ligand receptor pairs is not unusual. Additionally, the SNPs examined for the erythrocyte genes in the whole genome data were primarily rare variant loci, suggesting more population data is required or from the current available data there is limited variation in these genes giving credence to the observation predominantly >90% one haplotype except for Gyp A and singleton SNP loci. Consequently, in most ligand-receptor pairs there is an indication that the genes show evidence of the same directionality of Tajima's D statistic, for instance EBL-1-Gyp B were both negative.

However, the loci that show positive values (EBA175, *p* > .05 and MSP1, p > .05), are of particular interest as they may provide important functional information and identify regions potentially associated with disease. EBA175 region II has been shown to be immunogenic (Richards et al., 2010) and the several polymorphisms in it have been attributed to immune selection pressure (Baum et al., 2003; Verra et al., 2006). However, since Gyp A exon 2 was the most diverse receptor with the greatest variation between samples, the impact of the multiple polymorphisms in Gyp A exon 2 on EBA175 cannot be ruled out. The second merozoite ligand that showed a Tajima's D value >1 was MSP1. Previous studies have demonstrated that the upstream region closer to the N-terminal end of MSP1 is under selection (Hughes, 1992; Escalante et al., 1998). This is likely to be due to its abundance on the merozoite surface and the fact that it is a target of host immunity (Egan et al., 1996). These residues may be involved in escaping host immunity, since the receptor (band 3) regions interacting with these proteins had only one polymorphic site.

Our main observation of a negative summary statistic was evident in BSG exon 3. Though it is not expressed in BSG-S, the abundant isoform on erythrocytes, it is specific to the BSG-L isoform (the retinal form) which is only expressed in photoreceptor neurons (Ochrietor et al., 2003; Pablo and Ochrietor, 2013). This specific and limited expression, suggests that the BSG-L isoform may play a critical role in the retina. Thus, the acquisition of mutations in this region of the gene is likely to be minimised to preserve its highly specialised function. Nevertheless, the regions (exons 5 and 6) expressed on erythrocytes contained a limited number of polymorphic sites and in exon 5 there were rare synonymous and non-synonymous, L90F, variants identified in 1 individual each. Exon 6 contained one high frequency polymorphic synonymous site and a singleton non-synonymous site. In a previous study, a different amino acid change (L90P) in the rare variant codon showed a lower binding affinity for recombinant Rh5 protein (Crosnier et al., 2011), providing support to the idea that mutations are rare in this protein since they may alter protein function in a way that is detrimental. Additionally, the BSG-S isoform is expressed in many cell types throughout the body (Spring et al., 1997), it is therefore also possible that it cannot acquire multiple mutations as this may alter its function and impact many cellular processes. The importance of the interaction of Rh5 with BSG for invasion (Crosnier et al., 2011), the lack of an Rh5 knockout (Baum et al., 2009) and evidence demonstrating that Rh5 antibodies substantially inhibit invasion (Bustamante et al., 2013), emphasize its functional importance and may explain the few SNPs we observed. Rh5 contained a total of 7 non-synonymous SNPs, which may also be due to the limited number of polymorphisms it encounters on interaction with BSG and its relatively low immunogenicity (Douglas et al., 2011; Tran et al., 2014). Apart from one high frequency amino acid change located in the binding region with BSG, the other three high frequency amino acid changes are more likely to be the result of an immune escape mechanism, since they do not appear to be located in the region involved in the interaction with Basigin.

EBL-1 region II has previously been shown to be under purifying selection (Verra et al., 2006) and has been described as a recent pseudogene due to the stop codon in region II. We observed the same frequency of the 185 stop codon (due to the 5T nucleotide insertion) as Githui et al. (2010) from their analysis of 47 different Kenyan sequences, of 72%. From a smaller study done in Kilifi at a later time point (2008) and a smaller number of samples (19), the frequency of the 185 stop codon was 58% and 16% for stop codon 39 (Ochola-Oyier et al., 2016). In region II, we identified a different stop codon (664) in 2 samples compared to the Verra et al. (2006) study that identified stops in codons 253 and 469 in two and one sample, respectively. This study therefore complements other studies (Verra et al., 2006; Githui et al., 2010; Ochola-Oyier et al., 2016) providing further evidence that there is a loss in ebl-1 function and that there is a predominance of the 5 T insertion in the Kilifi population. It also demonstrates that the merozoite invasion of erythrocytes is a redundant process that uses multiple ligand-receptor pathways and it indicates that it may not be a major ligand for invasion. Its receptor, Gyp B, showed <−1 Tajima's D suggesting that this gene also limits the acquisition of multiple mutations, potentially to preserve its function, which is not yet known. Moreover, it is a locus that contributes to variation in this gene cluster through recombination and gene conversion events, generating hybrids with Gyp A (Baum et al., 2002; Wang et al., 2003; Ko et al., 2011; Leffler et al., 2017), since it arose through a duplication event of Gyp A but lacks exon 3 through a splice site mutation (Kudo and Fukuda, 1989). A recent study showed wide variation in the levels of Gyp B transcripts in Beninese children (Dankwa et al., 2017). It is perhaps such variation that would warrant the presence of stop codons in EBL-1, since in individuals with low Gyp B transcript levels the parasite would require a different ligand-receptor interaction for the invasion of the erythrocyte.

An analysis of multiple erythrocyte receptor genes, which primarily have a negative summary statistic similar to a large majority of the human genome and limited variation from the 1000 whole genome analysis, prompted a closer examination into the function of these genes, BSG, CR1 and band 3. They have specific functions, from the transport of nutrients and induction of matrix metalloproteinases (Muramatsu, 2016), to serving as the immune adherence receptor for complement (C3b/C4b) coated antigens (Java et al., 2015), and being the most abundant protein and major anion HCO_3_^−^Cl^−^ exchanger in the erythrocyte membrane (Reithmeier et al., 2016), respectively. Gyp A which appears to primarily maintain the negatively charged glycocalyx to prevent erythrocyte aggregation (Poole, 2000), has potentially been modified by its interactions with parasites, bacteria and viruses. There is also evidence that BSG interacts with bacteria and viruses (Muramatsu, 2016), however due to its functional importance as well as that of CR1 and band 3, there is less diversification of these genes compared to Gyp A. This study like three previous studies (Gagneux and Varki, 1999; Baum et al., 2002; Ko et al., 2011), is consistent with the suggestion that the erythrocytes act as decoys for pathogens to be cleared and taken away from more important cells in the body, thus the impact of the parasite on erythrocyte receptors appears to be minimal since their primary role is to maintain their function in the erythrocyte. Furthermore, the examination of the merozoite ligands and erythrocyte receptor pairs in a population with severe malaria, is consistent with previous studies that investigated either the ligand or receptor alone. Thus, it provides similar and unifying results of the observed negative or positive Tajima's D values, suggesting that it is also representative of the wider population and not the result of specific ligand-receptor populations of those assessed in this study.

## 5 Conclusions

Though a large majority of the regions analysed in the *P. falciparum* merozoite ligand – human erythrocyte receptor gene pairs appeared to be neutral, four pairs are highlighted as interesting due to either >1 and <−1 Tajima's D value in one or both gene pairs. EBA175 and Gyp A, EBA175 had the highest positive Tajima's D value >1 and it is the dominant ligand of the sialic-acid dependent invasion pathway for the parasite (Duraisingh et al., 2003), while Gyp A is potentially a receptor for other pathogens due to its abundance on the erythrocyte surface. Similarly, MSP1–33, which also had a > 1 Tajima's D, and its receptor band 3 are abundant proteins on the surface of the merozoite and erythrocyte, respectively, which mediate the initial merozoite-erythrocyte interaction for invasion (Cowman et al., 2017). EBL-1 and Gyp B were the pair with both negative Tajima's D values <−1 and the studied population contained predominantly the non-functional EBL-1 variant. They are both involved in an alternative sialic-acid dependent invasion pathway. The Rh5 and Basigin (<−1 Tajima's D) interaction has been described as a sialic-acid independent pathway (Baum et al., 2009), and both genes have important cellular functions, Rh5's critical role in invasion and BSG's role in multiple cellular processes. Since the merozoite invasion of erythrocytes is a critical part of disease pathology and a target of vaccine development, the four gene pairs are highlighted as interesting targets for interventions. Further work is required to understand the impact of the genetic variation on their interaction with erythrocyte receptors and host immunity.

The following are the supplementary data related to this article.

Supplementary Table 1 PCR conditions and primer sequences for the gene regions analysed in the six erythrocyte and merozoite genes. Supplementary Table 1

Supplementary Table 2 Summary of whole genome Tajima's D values >1 from 3 previously published African parasite populations. Supplementary Table 2

## Author contributions

LIO-O conceived, designed and oversaw the study, generated and analysed sequence data, performed the neutrality indices analysis, interpreted the data and wrote the manuscript, KW collated and analysed all the sequence data and performed the neutrality indices analysis, IO generated and analysed sequence data and reviewed the manuscript, CO, JPM & FKN generated and analysed sequence data, AM conducted the PCR-RFLP for DARC and PCRs for *P.vivax*, KM conceived the study and reviewed the manuscript. All authors read and approved the final manuscript.

## Data accessibility

The DNA sequence data for both the erythrocyte receptors and merozoite ligand genes were deposited in Genbank and are available under the accession codes, EBA140:MG023845-MG023885, EBA175:MG024023-MG024052, EBL1-I:MG023740-MG023765, EBL1-II:MG023362-MG023399, MSP1-19:MG024228-MG024276, MSP1-42:MG024420-MG024466, Rh4:MG023657-MG023701, Rh5:MG023323-MG023361, Band_3_Exon_17:MG024277-MG024347, Band_3_Exon_18:MG024088-MG024158, Basigin_Exon_3: MG023908-MG023968, Basigin_Exon_5: MG024159-MG024227, Basigin_Exon_6:MG023400-MG023465, CR1_Exon_4:MG023466-MG023539, CR1_Exon_5: MG023574- G023656, GYA_Exon_2:MG023969-MG024022, GYPA_Exon_4:MG024370-MG024419, GYPA_Exon_5:MG023766-MG023814, GYPB_ Exon_2: MG024053-MG024087, GYPB_Exon_3:MG023540 - MG023573, GYPB_Exon_4:MG024348-MG024369, GYPC_Exon_2:MG023815-MG023844 and GYPC_Exon_3:MG023886-MG023907.

## Competing interests

The authors declare that they have no competing interests.
