# Supplementary material for: Few Plasmodium falciparum merozoite ligand and erythrocyte receptor pairs show evidence of balancing selection
Source: Infect Genet Evol. 2019 Apr;69:235–45. doi: 10.1016/j.meegid.2019.02.004 (PMC6403450; doi:10.1016/j.meegid.2019.02.004)
Supplement: Supplementary Table 2 — Summary of whole genome Tajima's D values >1 from 3 previously published African parasite populations. [file mmc2.docx]

Supplementary Table 2

| **Gene ID** | **Gene Name** | **Tajima’s D** | **Country** | **Ref** |
| --- | --- | --- | --- | --- |
| PF3D7_1001100 | Acyl-CoA binding protein, isoform 1 | 1.83 | Gambia | Ngwa et al 2012 |
| PF3D7_0103600 | Adenosine triphosphate–dependent RNA helicase; putative | 1.03 | Malawi | Ocholla et al 2014 |
| PF3D7_1004800 | ADP/ATP carrier protein, putative | 2.34 | Guinea | Mobegi et al 2014 |
| PF3D7_1004800 | ADP/ATP carrier protein, putative | 2.2 | Gambia | Mobegi et al 2014 |
| PF3D7_1004800 | ADP/ATP carrier protein, putative | 2.6 | Gambia | Ngwa et al 2012 |
| PF3D7_1133400 | Apical membrane antigen 1 (AMA1) | 1.95 | Gambia | Ngwa et al 2012 |
| PF3D7_1133400 | Apical membrane antigen 1 (AMA1) | 1.72 | Malawi | Ocholla et al 2014 |
| PF3D7_1133400 | Apical membrane antigen 1 (AMA1) | 1.7 | Gambia | Mobegi et al 2014 |
| PF3D7_1133400 | Apical membrane antigen 1 (AMA1) | 1.45 | Guinea | Mobegi et al 2014 |
| PF3D7_0103600 | ATP-dependent RNA helicase, putative | 1.65 | Gambia | Ngwa et al 2012 |
| PF3D7_1126100 | Autophagy-related protein 7; putative | 1.57 | Malawi | Ocholla et al 2014 |
| PF3D7_0709300 | Cg2 protein | 1.96 | Malawi | Ocholla et al 2014 |
| PF3D7_0221000 | Conserved Plasmodium falciparum protein family | 1.93 | Gambia | Ngwa et al 2012 |
| PF3D7_0104100 | Conserved plasmodium membrane protein, unknown function | 1.16 | Guinea | Mobegi et al 2014 |
| PF3D7_0104100 | Conserved plasmodium membrane protein, unknown function | 1.05 | Gambia | Mobegi et al 2014 |
| PF3D7_0104100 | Conserved Plasmodium membrane protein, unknown function | 1.49 | Malawi | Ocholla et al 2014 |
| PF3D7_0710200 | Conserved plasmodium protein | 1.41 | Gambia | Mobegi et al 2014 |
| PF3D7_0710200 | Conserved plasmodium protein | 1.32 | Guinea | Mobegi et al 2014 |
| PF3D7_0710000 | Conserved Plasmodium protein, unknown function | 2.52 | Malawi | Ocholla et al 2014 |
| PF3D7_0710200 | Conserved Plasmodium protein, unknown function | 1.94 | Malawi | Ocholla et al 2014 |
| PF3D7_0630800 | Conserved Plasmodium protein, unknown function | 1.83 | Malawi | Ocholla et al 2014 |
| PF3D7_0729700 | Conserved, unknown function | 1.97 | Gambia | Ngwa et al 2012 |
| PF3D7_0710200 | Conserved, unknown function | 1.97 | Gambia | Ngwa et al 2012 |
| PF3D7_0831600 | Cytoadherence linked asexual protein 8 (CLAG8) | 2.79 | Gambia | Ngwa et al 2012 |
| PF3D7_0220800 | Cytoadherence linked asexual protein 2 (CLAG2) | 1.61 | Gambia | Ngwa et al 2012 |
| PF3D7_0302200 | Cytoadherence linked asexual protein 3.2 (CLAG3.2) | 2.02 | Gambia | Ngwa et al 2012 |
| PF3D7_0113800 | DBL containing protein, unknown function | 1.59 | Gambia | Ngwa et al 2012 |
| PF3D7_0113800 | DBL-containing protein, unknown function | 1.36 | Guinea | Mobegi et al 2014 |
| PF3D7_0113800 | DBL-containing protein, unknown function | 1.15 | Gambia | Mobegi et al 2014 |
| PF3D7_0630300 | DNA polymerase epsilon, catalytic subunit a, putative | 1.15 | Malawi | Ocholla et al 2014 |
| PF3D7_1149600 | DnaJ protein; putative | 1.59 | Malawi | Ocholla et al 2014 |
| PF3D7_1035700 | Duffy binding-like merozoite surface protein (DBLMSP) | 1.81 | Gambia | Ngwa et al 2012 |
| PF3D7_1035700 | Duffy binding-like merozoite surface protein (DBLMSP) | 1.01 | Malawi | Ocholla et al 2014 |
| PF3D7_1035700 | Duffy binding-like merozoite surface protein (DBLMSP) | 2.48 | Gambia | Mobegi et al 2014 |
| PF3D7_1035700 | Duffy binding-like merozoite surface protein (DBLMSP) | 1.33 | Guinea | Mobegi et al 2014 |
| PF3D7_1036300 | Duffy binding-like merozoite surface protein 2 (DBLMSP2) | 2.5 | Gambia | Mobegi et al 2014 |
| PF3D7_1036300 | Duffy binding-like merozoite surface protein 2 (DBLMSP2) | **3.31** | Gambia | Ngwa et al 2012 |
| PF3D7_1036300 | Duffy binding-like merozoite surface protein 2 (DBLMSP2) | **3.26** | Malawi | Ocholla et al 2014 |
| PF3D7_1036300 | Duffy binding-like merozoite surface protein 2 (DBLMSP2) | **2.68** | Guinea | Mobegi et al 2014 |
| PF3D7_0113800 | Duffy binding-like–containing protein, unknown function | 1.24 | Malawi | Ocholla et al 2014 |
| **PF3D7_0731500** | **Erythrocyte binding antigen-175** | **1.56** | **Malawi** | **Ocholla et al 2014** |
| PF3D7_0720400 | Ferrodoxin reductase-like protein | 1.82 | Gambia | Ngwa et al 2012 |
| PF3D7_0720400 | Ferrodoxin reductase-like protein | 1.29 | Gambia | Mobegi et al 2014 |
| PF3D7_0720400 | Ferrodoxin reductase-like protein | 1.06 | Guinea | Mobegi et al 2014 |
| PF3D7_0420200 | Holo-(acyl-carrier protein) synthase, putative | 2.02 | Gambia | Mobegi et al 2014 |
| PF3D7_0420200 | Holo-(acyl-carrier protein) synthase, putative | 1.3 | Guinea | Mobegi et al 2014 |
| PF3D7_0420200 | holo-[acyl-carrier-protein] synthase, putative | 2.27 | Gambia | Ngwa et al 2012 |
| PF3D7_1035400 | Merozoite surface protein 3 | 1.37 | Malawi | Ocholla et al 2014 |
| PF3D7_0511300 | MORN repeat protein, putative | 1.63 | Gambia | Ngwa et al 2012 |
| PF3D7_0321200 | N-acetylglucosamine-1-phosphate transferase, putative | 1.68 | Gambia | Mobegi et al 2014 |
| PF3D7_0321200 | N-acetylglucosamine-1-phosphate transferase, putative | 1.65 | Guinea | Mobegi et al 2014 |
| PF3D7_0824400 | Nucleoside transporter 2 | 1.7 | Gambia | Ngwa et al 2012 |
| PF3D7_0710400 | Nucleotide excision repair protein | 1.25 | Guinea | Mobegi et al 2014 |
| PF3D7_0710400 | Nucleotide excision repair protein | 1.07 | Gambia | Mobegi et al 2014 |
| PF3D7_0101300 | Pfmc-2TM Maurer's cleft two transmembrane protein | 2.04 | Gambia | Ngwa et al 2012 |
| PF3D7_1100800 | Pfmc-2TM_11.1 Maurer's cleft protein | 2.16 | Gambia | Ngwa et al 2012 |
| PF3D7_0701900 | Plasmodium exported protein; unknown function | 1.82 | Gambia | Ngwa et al 2012 |
| PF3D7_0425400 | Plasmodium exported protein (PHISTa), unknown function | 2.06 | Malawi | Ocholla et al 2014 |
| PF3D7_1253100 | Plasmodium exported protein (PHISTa), unknown function | 2.49 | Gambia | Ngwa et al 2012 |
| PF3D7_0201600 | PHISTb domain-containing RESA-like protein 1 | 1.84 | Gambia | Ngwa et al 2012 |
| PF3D7_0221000 | Plasmodium exported protein, unknown function | 1.98 | Malawi | Ocholla et al 2014 |
| PF3D7_0221000 | Plasmodium-exported protein, unknown function | 2.03 | Guinea | Mobegi et al 2014 |
| PF3D7_0221000 | Plasmodium-exported protein, unknown function | 1.8 | Gambia | Mobegi et al 2014 |
| PF3D7_0114500 | Plasmodium exported protein (hyp10) | 2.76 | Gambia | Ngwa et al 2012 |
| PF3D7_0114500 | Plasmodium-exported protein (hyp10) | 1.27 | Gambia | Mobegi et al 2014 |
| PF3D7_0114500 | Plasmodium-exported protein (hyp10) | 1.04 | Guinea | Mobegi et al 2014 |
| PF3D7_1253100 | Plasmodium-exported protein (PHISTa) | **2.82** | Gambia | Mobegi et al 2014 |
| PF3D7_1253100 | Plasmodium-exported protein (PHISTa) | 2.11 | Guinea | Mobegi et al 2014 |
| PF3D7_0201600 | PHISTb domain-containing RESA-like protein 1 (RLP) | 2.46 | Guinea | Mobegi et al 2014 |
| PF3D7_0201600 | PHISTb domain-containing RESA-like protein 1 (RLP) | 2.07 | Gambia | Mobegi et al 2014 |
| PF3D7_0601500 | Plasmodium-exported protein (PHISTb) | 1.64 | Gambia | Mobegi et al 2014 |
| PF3D7_0601500 | Plasmodium-exported protein (PHISTb) | 1.05 | Guinea | Mobegi et al 2014 |
| PF3D7_1319700 | Ser/thr protein phosphatase 2A subunit | 1.63 | Gambia | Ngwa et al 2012 |
| PF3D7_0508800 | Single-stranded DNA-binding protein (SSB) | 1.1 | Guinea | Mobegi et al 2014 |
| PF3D7_0508800 | Single-stranded DNA-binding protein (SSB) | 1.07 | Gambia | Mobegi et al 2014 |
| PF3D7_1301800 | Surface-associated interspersed protein 13.1 (SURFIN 13.1) | 1.42 | Gambia | Mobegi et al 2014 |
| PF3D7_1301800 | Surface-associated interspersed protein 13.1 (SURFIN 13.1) | 1.2 | Guinea | Mobegi et al 2014 |
| PF3D7_0424400 | Surface-associated interspersed protein 4.2 (SURFIN 4.2) | 1.85 | Malawi | Ocholla et al 2014 |
| PF3D7_0830800 | Surface-associated interspersed protein 8 (SURFIN 8) | 1.85 | Gambia | Ngwa et al 2012 |
| PF3D7_0516300 | Transfer RNA pseudouridine synthase; putative | 1.39 | Malawi | Ocholla et al 2014 |

Tajima’s D values bold and underlined indicate the genes with the highest Tajima’s D value in each population. Text in bold indicates EBA175, which was only identified in a Malawian population with a >1 Tajima’s D.
